# Dynamic Changes in Antimicrobial Resistance in Fecal *Escherichia coli* from Neonatal Dairy Calves: An Individual Follow-Up Study

**DOI:** 10.3390/ani10101776

**Published:** 2020-10-01

**Authors:** Sang-Ik Oh, Seungmin Ha, Jae-Hee Roh, Tai-Young Hur, Jae Gyu Yoo

**Affiliations:** National Institute of Animal Science, Rural Development Administration, 1500 Kongjwipatjwi-ro, Wanju 55365, Korea; ohsangik@korea.kr (S.-I.O.); justusha@korea.kr (S.H.); snowida@korea.kr (J.-H.R.); tyohur@korea.kr (T.-Y.H.)

**Keywords:** antimicrobial resistance, antimicrobial resistance gene, colostrum, fecal *Escherichia coli*, neonatal dairy calf

## Abstract

**Simple Summary:**

Antimicrobial resistance in food animals is a global public health concern. In dairy farms, young calves typically carry high levels of antimicrobial-resistant (AMR) *Escherichia coli*, and may act as a potential reservoir. Fecal *E. coli* were isolated and tested for susceptibilities to eight common antimicrobials from 19 newborn dairy calves using individual follow-up at daily and weekly intervals. Shedding of AMR *E. coli* first appeared at 2–3 days after birth. The majority of fecal *E. coli* from neonatal calves (≤28 days old) were resistant to streptomycin, sulfisoxazole, and tetracycline. A tetracyclines-associated resistance gene (*tetB*) was predominant in the fecal *E. coli* from neonatal calves, and was also detected in maternal colostrum samples from the mothers of the tested calves. These results suggest the potential of antimicrobial resistance genes being shared between neonatal calves and their mothers’ colostrum. Neonatal calves with a history of treatment with ceftiofur also shed AMR *E. coli* resistant against beta-lactams. Moreover, these results provide new insights for controlling the spread of antimicrobial resistance on dairy farms.

**Abstract:**

The prevalence of antimicrobial-resistant (AMR) *Escherichia coli* is typically higher in the feces of young dairy calves than in the feces of older cattle; however, the underlying factors contributing to this difference are poorly understood. In this study, AMR fecal *E. coli* from neonatal calves were characterized both at phenotypic and genotypic levels by individual follow-up sampling. Antimicrobial resistance profiles of *E. coli* isolates from the maternal colostrum were also determined. Most of the fecal AMR *E. coli* emerged in the calves at 2–3 days of age. The *tetB* was the most prevalent resistance gene detected among AMR fecal *E. coli* from <7-day-old calves, and was also detected in two isolates from the maternal colostrum. Weekly sampling revealed changes in the phenotype of AMR fecal *E. coli* as the calves aged. More than half of the fecal *E. coli* isolates acquired additional resistance to beta-lactams by 21–28 days of age, and minimum inhibitory concentrations were higher in ceftiofur-exposed calves than in unexposed calves. Our findings reveal the dynamic changes in AMR fecal *E. coli* from neonatal calves, and suggest that the feeding of colostrum and ceftiofur administration contribute to the higher prevalence of AMR *E. coli* in young dairy calves.

## 1. Introduction

Antimicrobial resistance is a global challenge in both food-producing animals and humans [1,2,3]. The widespread overuse and misuse of antibiotics in animal husbandry and human medicine have caused the emergence of multidrug-resistant bacteria, which could result in treatment failure [2]. Although the increased prevalence of resistant pathogenic bacteria is of prime concern, commensal bacteria, especially non-pathogenic *Escherichia coli*, are also considered as a potential reservoir of antimicrobial resistance [4]. Furthermore, the horizontal transfer of antimicrobial resistance genes (ARGs) is possible between commensal bacteria and pathogenic strains [1,5].

In dairy cattle, the prevalence of antimicrobial-resistant (AMR) *E. coli* and ARGs is age-dependent, with a higher prevalence detected in earlier stages of life, especially between 2–4 weeks old dairy calves [5,6,7]. Several studies have evaluated the factors contributing to this high prevalence of AMR *E. coli* in young dairy calves, and some studies have suggested the milk diet as an important risk factor [1,6,7,8,9,10]. Supplementation of calf milk with antimicrobials, incidence of diarrhea, use of feed additives with biocides and heavy metals, and vitamin supplements has also been suggested to contribute to the high AMR prevalence in young calves [1,6,8,9]. Previous studies have proposed that maternal colostrum, being the first feed, may be an important vehicle for AMR *E. coli* in neonatal calves [1,11,12]. A recent study revealed that fecal *E. coli* from 2-day-old calves share appropriately 90% of their ARGs with the *E. coli* in their maternal colostrum, thereby suggesting that the colostrum serves as a vector for ARGs transmitted to dairy calves [1]. In addition, Doyle et al. [13] suggested that the presence of feces on the teat surface is the most abundant contributor to the raw milk microbiota, indicating that AMR *E. coli* and their ARGs in the colostrum are environment-specific contaminants. However, most of these previous studies focused on animals without considering the detailed prior history of antibiotic use and clinical signs of disease [6,9,10]. Moreover, few studies have specifically investigated AMR fecal *E. coli* in neonatal calves with individual follow-up of short term intervals from the day of birth, which makes it difficult to specify when the AMR fecal *E. coli* emerges in newborn calves [5]. As a result, the epidemiology and dynamic changes of AMR fecal *E. coli* in neonatal calves are not fully understood.

To address these knowledge gaps, we evaluated changes in the antimicrobial resistance patterns of fecal *E. coli* isolated from newborn dairy calves using individual follow-up fecal sampling from the day of birth (once daily and weekly follow-up sampling). This detailed sampling approach with corresponding information of the tested animals is expected to help discriminate the precise timing of AMR fecal *E. coli* shedding from neonatal calves. Furthermore, we focused on investigating the role of two risk factors (colostrum and antibiotic use) which are related to the emergence timing of AMR fecal *E. coli* in neonatal calves. The study outcomes offer novel insights into the potentially influential factors determining a high AMR prevalence in young dairy calves.

## 2. Materials and Methods 

### 2.1. Animals

The study was performed at a large breeding dairy farm in Korea. A total of 29 newborn dairy calves who were born between October 2019 and April 2020 and were available for analysis were included in this study (Table 1). Newborn calves were separated from their dams within a few hours after birth and housed in a separate hutch. All neonatal calves were fed their respective dams’ colostrum within the first 12 h after birth until 3 days post-birth. After 3 days, all calves were fed whole milk until 28 days. The diet was supplemented with concentrated feed and Timothy hay as of between day 7 and 28 onward, respectively. The health and welfare of the calves included in the study were individually supervised by a designated veterinarian, who provided the detailed medical history for the calves and their dams. All experiments were approved by the Institutional Animal Care and Use Committee at the National Institute of Animal Science, Korea (approval number: NIAS 2019-369).

### 2.2. Sample Collection

**Figure 1:**
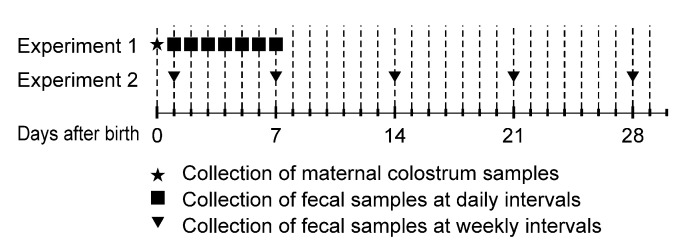
Sampling scheme for experiments 1 and 2. Neonatal dairy calves were sampled once a day for 7 days (*n* = 10) or once a week for 28 days (*n* = 19), and maternal colostrum samples (*n* = 6) from corresponding dams were obtained.

The detailed scheme for sampling is shown in Figure 1. In experiment 1, all of the dairy calves (*n* = 10) which were born during this experiment period were subjected to once-daily sampling 1 to 7 days after birth. Among these 10 calves, six maternal colostrum samples (15 mL each) from the corresponding dam were collected prior to feeding. Colostrum was harvested aseptically after predipping, wiping, and drying the teats, and then the samples were collected into sterile containers. In experiment 2, individual fecal samples were collected from 19 newborn dairy calves (79.2% of calves born during this experiment period) within the first day after birth and then at weekly intervals for up to 4 weeks. The fecal samples were obtained by swabbing the rectum with a sterile cotton swab. After collection, the samples were immediately sent to the laboratory in thermal bags with ice packs and stored at 4 °C until use. The bacteria were isolated from the collected samples within one day, and then the fecal samples were stored at −80 °C until further study.

### 2.3. Treatment History

The farm where the study was conducted commonly uses ceftiofur for treating respiratory tract infections, hoof disease, mastitis, and neonatal calf diarrhea (NCD). The history of clinical diseases and antibiotic use for each sampled calf and their dams is shown in Table 1. None of the 10 calves in experiment 1 received any antibiotics during the sampling period, and the dams of the tested calves did not receive antibiotics for the 1 year period before the birth of the calf. However, in experiment 2, five calves (calf nos. 23–27) showed signs of NCD, and were thus treated with ceftiofur; three dams of the sampled calves (calf nos. 27–29) received ceftiofur to treat hoof disease, foot rot, and mastitis, respectively. This detailed medical history provided an opportunity to directly test the impact of antibiotic exposure on the prevalence of AMR *E. coli*. Overall, 7 calves (36.8%) were assigned to the antibiotic exposed group and 12 (63.2%) were assigned to the non-exposed group for this comparison.

### 2.4. E. coli Isolation and DNA Extraction

The rectal swabs and maternal colostrum samples were cultured on MacConkey agar plates (Kisan Pharm, Seoul, Korea) and incubated aerobically at 37 °C for 24 h. Three pink colonies of different morphologies suspected as *E. coli* were selected and restreaked onto eosin-methylene blue (EMB) agar plates (Kisan Pharm), followed by incubation for 24 h at 37 °C. Next, the blue-black colonies with a metallic green sheen on EMB agar plates were selected and streaked onto 5% sheep blood agar plates. *E. coli* isolates were confirmed by biochemical identification using the Sensititre system (TREK Diagnostic System, East Grinstead, UK) with commercial GN plates, and also by 16S rRNA sequencing. Genomic DNA was extracted from the confirmed *E. coli* isolates using the QIAamp DNA Mini Kit (Qiagen, Hilden, Germany) according to the manufacturer’s instructions.

### 2.5. Identification of Antimicrobial Resistance Genes

ARGs were detected using previously published primers for detecting genes coding for resistance to streptomycin (*strA* and *strB*) [14], sulfonamide (*sulI* and *sulII*) [15], and tetracycline (*tetA* and *tetB*) [16]. The primers used were as follows [14,15,16]: *strA* [forward primer (F), 5′-CTTGGTGATAACGGCAATTC-3′; reverse primer (R), 5′-CCAATCGCAGATAGAAGGC-3′], *strB* (F, 5′-ATCGTCAAGGGATTGAAACC-3′; R, 5′-GGATCGTAGAACATATTGGC-3′), *sulI* (F, 5′-GTGACGGTGTTCGGCATTCT-3′; R, 5′-TCCGAGAAGGTGATTGCGCT-3′), *sulII* (F, 5′-CGGCATCGTCAACATAACCT-3′; R, 5′-TGTGCGGATGAAGTCAGCTC-3′), *tetA* (F, 5′-GGCCTCAATTTCCTGACG-3′; R, 5′-AAGCAGGATGTAGCCTGTGC-3′), and *tetB* (F, 5′-GAGACGCAATCGAATTCGG-3′; R, 5′-TTTAGTGGCTATTCTTCCTGCC-3′). Amplification was performed on a BioRad T100 thermocycler (Bio-Rad Laboratories Ltd., Hercules, CA, USA) under previously described conditions [13,14,15]. In brief, the annealing temperature and fragment size were as follows: *strA* (53 °C and 548 bp), *strB* (53 °C and 509 bp), *sulI* (68 °C and 779 bp), *sulII* (66 °C and 721 bp), *tetA* (55 °C and 372 bp), and *tetB* (55 °C and 228 bp). The amplified products were resolved by electrophoresis on 1.5% agarose gels, stained with RedSafe nucleic acid staining solution (iNtRON Biotechnology, Seongnam, Korea), and visualized with an ultraviolet transilluminator (Bio-Rad Laboratories Ltd., Hercules, CA, USA).

### 2.6. Antimicrobial Susceptibility Test

Minimum inhibitory concentrations (MICs) of *E. coli* were determined by the standard microbroth dilution method using the Sensititre system (TREK Diagnostic System, East Grinstead, UK) with antimicrobial testing plates containing the following eight antimicrobials: amoxicillin-clavulanic acid (AMO, range tested: 1–64 μg/mL), ampicillin (AMP, range tested: 1–128 μg/mL), cefoxitin (CFX, range tested: 0.5–64 μg/mL), ceftiofur (TIO, range tested: 0.25–16 μg/mL), streptomycin (STR, range tested: 8–256 μg/mL), sulfisoxazole (FIS, range tested: 8–512 μg/mL), tetracycline (TET, range tested: 1–256 μg/mL), and trimethoprim-sulfamethoxazole (STX, range tested: 0.0625–8 μg/mL). *E. coli* strains ATCC 25922 (American Type Culture Collection (ATCC; Manassas, VA, USA)) and *Enterococcus faecalis* ATCC 29212 were used as quality control strains. MICs were interpreted according to guidelines of the CLSI (Clinical and Laboratory Standards Institute) [17] or DANMAP (Danish Integrated Antimicrobial Resistance Monitoring and Research Programme) [18] in the case of streptomycin, for which CLSI breakpoints were not available.

### 2.7. Statistical Analysis

Statistical analyses were performed using SPSS version 25.0 (IBM, Armonk, NY, USA). A simple regression model was applied to determine whether there was a significant difference in antibiogram (ABG) patterns from fecal *E. coli* according to the calves’ age. The mean differences in the MIC values were analyzed using repeated-measures analysis of variance, with group (antibiotic exposure) and time as the main effects, and an additional group-by-time interaction term. Results with *p* < 0.05 were considered statistically significant.

## 3. Results

### 3.1. Experiment 1

**Figure 2:**
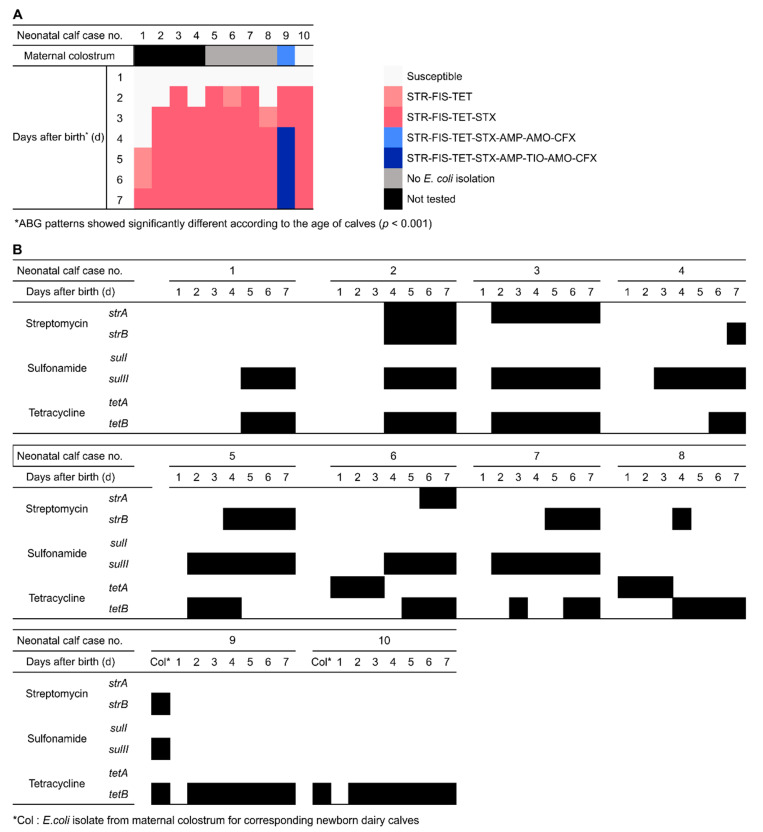
Characterization of resistance to eight antimicrobials in *E. coli* isolated from newborn dairy calves (*n* = 10) within the first day after birth and at daily intervals for up to 7 days. (**A**) Antibiogram (ABG) of fecal *E. coli* isolated from newborn calves. Each box represents a single isolate on a given sampling day. Colors represent ABG patterns, and unfilled boxes indicate that the isolates were susceptible to all tested antimicrobials. Black boxes indicate that no isolates were obtained from maternal colostrum samples. (**B**) Distribution of ARGs associated with streptomycin (*strA* and *strB*), sulfa-drugs (*sulI* and *sulII*), and tetracycline (*tetA* and *tetB*) in *E. coli* isolates from each calf and the corresponding maternal colostrum.

We analyzed the dynamic fecal *E. coli* ABG patterns in each dairy neonatal calf (*n* = 10) with daily sampling for one week (Figure 2A). The confirmed *E. coli* isolates (two or three isolates per sample) from the same fecal sample showed identical ABG patterns against the eight tested antimicrobials in this study. Thus, only one isolate per sample was selected for further analysis as a representative fecal *E. coli* from each calf per sampling day. A simple regression analysis showed that the ABG patterns of fecal *E. coli* were significantly different according to the age of the calves (adjusted *R*^2^ = 0.414, F = 48.075, *p* < 0.001). All 10 fecal *E. coli* isolates from 1-day-old calves were susceptible to the eight tested antimicrobials. AMR fecal *E. coli* started to shed from 2-day-old (*n* = 6, 60.0%), 3-day-old (*n* = 3, 30.0%), and 5-day-old (*n* = 1, 10.0%) calves. Among the 70 isolates, 50 fecal *E. coli* (71.4%) showed STR-FIS-TET or STR-FIS-TET-STX ABG patterns. Only one calf (no. 9) shed isolates with the STR-FIS-TET-STX-AMO-AMP-CFX-TIO ABG pattern from day 4 to 7 after birth. Only two *E. coli* strains were isolated from the six maternal colostrum samples collected in this study. One strain (from the dam of calf no. 9) was resistant to all tested antimicrobials except TIO, and the other (from the dam of calf no. 10) was susceptible to all of the tested antimicrobials.

The distributions of transmissible ARGs associated with resistance to streptomycin, sulfonamides, and tetracyclines in *E. coli*, isolates from the feces of newborn calves and their maternal colostrum are shown in Figure 2B. The *tetB* gene was the most prevalent resistance gene detected (*n* = 41, 75.9%) in 54 AMR fecal *E. coli* samples from calves, followed by *sulII* (63.0%), *strB* (25.9%), *strA* (24.1%), and *tetA* (5.6%). Although the distribution of ARGs in fecal *E. coli* isolates showed different patterns per calf, the overall rates of *strA*, *sulII*, and *tetB* were higher in 7-day-old calves. The *strB* gene was detected only in fecal *E. coli* from 4–7 days old calves, whereas *tetA* could be found in fecal *E. coli* of 1- and 3-day-old calves. In the maternal colostrum, one strain (isolated from the dam of calf no. 9) harbored *strB*, *sulII*, and *tetB*, and another (isolated from the dam of calf no. 10) had only *tetB*.

### 3.2. Experiment 2

**Figure 3:**
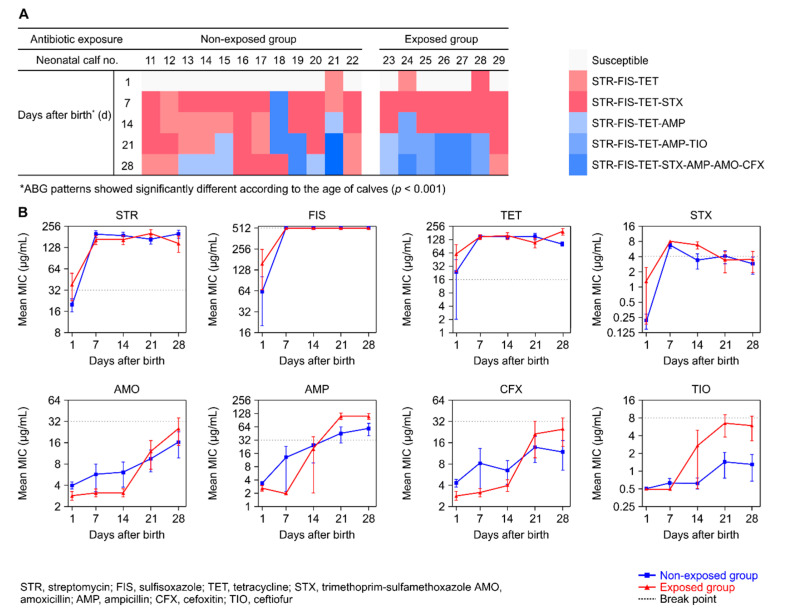
Characterization of resistance to eight antimicrobials in newborn dairy calves (*n* = 19) from the first day after birth and at weekly intervals for up to 4 weeks. (**A**) Antibiogram (ABG) of fecal *E. coli* isolated from newborn calves. Each box represents a single isolate on a given sampling day. Colors represent ABG patterns, and unfilled boxes indicate that isolates were susceptible to all tested antimicrobials. (**B**) Changes in mean minimum inhibitory concentration (MIC) values according to calf age. Blue lines indicate the mean MIC in cases with no prior treatment with antibiotics in calves and their dams, and red lines indicate MIC values in cases of prior ceftiofur use in calves or their dams. Error bars represent standard error of the mean.

We further investigated the ABG patterns for fecal *E. coli* isolated from neonatal calves (≤ 28 days old, *n* = 19) by performing once-weekly follow-up (Figure 3A). Overall, 79 fecal *E. coli* (83.2%) were resistant to at least one antimicrobial, and all isolates were resistant to STR, FIS, and TET. The ABG patterns for fecal *E. coli* isolates showed significant differences among calves according to their age distribution according to a simple regression analysis (adjusted *R*^2^ = 0.365, F = 53.517, *p* < 0.001). Most of the fecal *E. coli* isolates from 1-day-old calves (*n* = 16, 84.2%) were susceptible to all tested antimicrobials. The STR-FIS-TET and STR-FIS-TET-STX ABG patterns were found in 18 of 19 (94.7%) fecal samples from 7-day-old calves and in 15 of 19 (78.9%) samples from 14-day-old calves. The frequency of these ABG patterns decreased as the neonatal calves aged (21 days, 47.4%; 28 days, 36.8%). In contrast, the frequencies of beta-lactam–resistant (AMP, AMO, TIO, or CFX) fecal *E. coli* increased as the calves aged (5.3% for 7-day-old calves, 15.8% at 14 days, 52.6% at 21 days, and 63.2% at 28 days).

A comparative analysis of mean MIC values against eight antimicrobials between the non-exposed group (*n* = 12) and the exposed group (*n* = 7) is shown in Figure 3B. Mean MICs of STR, FIS, TET, and STX in both groups peaked during the period from birth to 7 days and were maintained at high levels until 28 days after birth. The MICs of these agents did not differ significantly between the two groups at any sampling point. The average MICs of beta-lactams (AMO, AMP, CFX, and TIO) in both groups increased gradually from days 1 to 28. Notably, we observed a significant effect of time on the MIC values of AMO (*p* < 0.001), AMP (*p* < 0.001), CFX (*p* = 0.014), and TIO (*p* = 0.001), and a significant group effect only on the MIC of TIO (*p* = 0.024). Furthermore, a significant time-by-group interaction was noted for the MIC values of AMP (*p* < 0.001) and TIO (*p* = 0.023).

## 4. Discussion

Young dairy calves generally show higher frequencies of AMR bacteria and ARGs than older cattle [5,19,20]. However, the epidemiology and determinants of these frequencies in young calves remain unclear, thereby limiting the development of strategies to reduce the burden of AMR bacteria on dairy farms. In this study, we determined the timing of fecal AMR *E. coli* shedding from neonatal dairy calves, and focused on two factors, colostrum and antibiotic use, to explain the elevated AMR rate in young calves.

Previous studies have suggested that AMR *E. coli* colonization occurs shortly after birth, typically between 2 and 4 weeks of age [5,6,7,20]. However, the specific timing of the emergence of AMR fecal *E. coli* in calves is unclear due to the lack of data based on daily sampling from the day of calf birth. The prevalence of AMR fecal *E. coli* in dairy calves may be affected by many factors, including the use of antibiotics, vitamin supplements, and maternal colostrum [1,6,8,9,10,20,21,22]. To clarify the precise underlying factors, it is important to establish the timing of AMR *E. coli* emergence in calves. Although the *E. coli* isolates included in this analysis might not completely represent the entire diversity of *E. coli* present in the gut of newborn calves, our results confirm that most fecal AMR *E. coli* isolates (90.0%) started being shed from neonatal calves at 2–3 days after birth, and these strains show simultaneous resistance to STR, FIS, and TET. These findings suggest that the occurrence of AMR fecal *E. coli* in newborn calves is not intrinsic and could be acquired shortly after birth. Moreover, as the calves (≤7 days old) and dams included in this experiment had never been administered antibiotics, including STR, FIS, and TET, the emergence time and early temporal phenotypic shifts of AMR in fecal *E. coli* in neonatal calves were intriguing. In our preliminary experiment, we determined that the *E. coli* isolates from solid food and water used for feeding calves were not resistant to the eight tested antibiotics. Moreover, the *E. coli* isolates from vaginal swab and fecal samples of the four dams (mothers of calf nos. 1–4) were susceptible for the tested antimicrobials. Therefore, based on our results and earlier reports [1,11,12], we suspected that the early prevalence of AMR *E. coli* in the calves is associated with the colostrum diet, which was provided from birth to day 3 in the farm.

Although we were only able to isolate *E. coli* strains from two maternal colostrum samples, one *E. coli* isolate showed similar ABG patterns to fecal *E. coli* from their corresponding calves aged 4–7 days. The calf fed the colostrum with *E. coli* susceptible to all antibiotics showed identical STR-FIS-TET-STX ABG patterns from 2 to 7 days of age. Although the number of isolates from colostrum samples was too small to establish a definitive conclusion, these results suggest the possibility of transmission in beta-lactams–resistant *E. coli* via feeding colostrum. However, these results could not explain the emergence of STR-FIS-TET-STX ABG *E. coli* from 2- and 3-day-old calves fed with colostrum. Therefore, another approach, such as genotypic characterization of AMR, is warranted to confirm the potential role of maternal colostrum on prevalence of AMR fecal *E. coli* in neonatal calves.

Metagenomics analyses of the gut resistome using fecal samples have recently been conducted to evaluate ARGs in cattle [1,23,24]. However, the results may not reflect all AMR phenotypes, as DNA from dead AMR *E. coli* could be released from the fecal sample. In this study, the presence of ARGs was investigated in *E. coli* isolates from feces of neonatal calves which showed various AMR phenotypes. The high frequency of the *tetB* efflux gene in the neonatal calves is consistent with previous studies’ results showing that 64.8% of TET-resistant strains from conventional and organic dairies possess *tetB* determinants, reflecting the frequent use of tetracycline drugs on the farm [22,25,26]. Tetracyclines were widely used for infection control and growth promotion worldwide, including Korea, until they were banned as feed additives, implying that the ARGs associated with TET have evolved in diverse dairy farm environments [24,25]. The current findings of a high prevalence of *tetB* in fecal *E. coli* from neonatal calves may also be related to the use of tetracyclines as feed additives until July 2011 (in accordance with Korean governmental policy), as the withdrawal of antibiotics does not immediately reduce the prevalence of AMR strains and ARGs [5,27]. Furthermore, *tetB* has been reported in other Gram-negative bacteria, implying that gene transfer between nonpathogenic and pathogenic bacteria could occur in dairy farms [28,29]. Therefore, continuous surveillance of transmissible ARGs of commensal bacteria in dairy calves is essential in dairy farms to prevent the emergence of AMR pathogenic bacteria. Although only two *E. coli* isolates were collected from maternal colostrum samples in this study, *tetB* genes were detected in both samples and the corresponding calf feces. Thus, the current findings suggest the potential for *tetB* transmission between colostrum and newborn calves, emphasizing the need for effective hygienic procedures in farm environments. Given that only two isolates from the colostrum were included in this study, it is essential to elucidate the actual role of the colostrum in acquiring AMR *E. coli* in newborn calves with a sufficiently large number of samples or with a metagenomics analysis of the colostrum. In addition, further studies should focus on finding other possible factors, including host-specific and environmental factors, responsible for the emergence of AMR fecal *E. coli* in young calves.

In weekly fecal sampling from neonatal calves (≤28 days old), all AMR fecal *E. coli* were resistant to STR, FIS, and TET, and the prevalence of STR-FIS-TET and STR-FIS-TET-STX ABG fecal *E. coli* decreased as calves aged. These findings are inconsistent with those of previous studies, which reported that the prevalence of STR-, FIS (or sulfadiazine)-, and TET-resistant fecal *E. coli* increases as calves age until 4 weeks after birth [5,10]. We hypothesize that the discrepancy in these results among studies could be attributed to the differences in the history of exposure to antibiotics in sampled calves. Indeed, a previous study suggested that the use of ceftiofur influences the emergence of resistance in *E. coli* and reduces antibiotic-susceptible strains shed from dairy calves [30]. In this study, seven sampled calves had a history of exposure to ceftiofur between 14 and 25 days of age, and fecal *E. coli* in these neonatal calves showed additional resistance to beta-lactams as they aged, especially after the age of 14 days. To elucidate the role of ceftiofur use for increasing AMR prevalence, we performed the comparative analysis of mean MIC values against eight antimicrobials between the non-exposed group (*n* = 12) and the exposed group (*n* = 7). Our results indicate that prior use of ceftiofur influenced the MICs of not only TIO but also AMP, implying that the repeated administration of specific antibiotics to farm animals could affect the MICs of other antibiotics in the same class. Despite previous studies showing that the effects of antibiotics on AMR are transient [6,30], our findings warn that AMR strains could become concentrated in feces and the dairy farm environment due to the shedding of AMR fecal *E. coli* from neonatal calves. Given that NCD is common in neonatal calves at around 2 weeks of age [31], it is necessary to establish appropriate antimicrobial stewardship practices for NCD treatment to prevent the further development of AMR in fecal *E. coli* from neonatal calves. Moreover, since pathogenic *E. coli* is known to be one of the major causes of NCD [32], further studies should investigate the prevalence of pathotypes in fecal *E. coli* from diarrheic calves, and their dynamic changes of ABG patterns regarding with the use of antibiotics. There is another limitation in this study that not all newborn calves—only 19 out of the 24 calves which were born during the weekly sampling period—were included in this study. Although the required sample size (≥18 newborn calve with a confidence level of 90% and an accepted error of 10%) could be achieved in this study, further studies with a large sample size are needed to generate a robust conclusion about the changes in antimicrobial resistance patterns of fecal *E. coli* from neonatal dairy calves.

## 5. Conclusions

In conclusion, our results show that AMR *E. coli* isolates begin to shed from 2–3 days old calves, and most of these isolates are resistant to STR, FIS, and TET. Furthermore, STR-FIS-TET or STR-FIS-TET-STX ABG patterns were most prevalent in neonatal calves (≤28 days old), and the patterns changed as calves aged. The distribution of ARGs was investigated in *E. coli* isolation from the feces of neonatal calves, and tetracyclines-associated resistance gene (*tetB*) was most prevalent. Although only two *E. coli* were analyzed in this study, *tetB* gene was also detected in *E. coli* isolates from the maternal colostrum samples, implying that the gene transmission mightoccur between neonatal calves and their maternal colostrum. Moreover, the current findings suggest that the use of antibiotics in neonatal calves could change the MIC values of multiple antimicrobials within the same class. Further studies are needed to determine the major risk factors to facilitate the development of strategies to reduce the burden of AMR *E. coli* on dairy farms.

## Figures and Tables

**Table 1:** Detailed information about 29 newborn calves included in this study.

Group	Calf No.	Breed	History of Clinical Signs and Antibiotic Use		
			Clinical Sign (Day after Birth)	Antibiotic Use (Day after Birth)	Antibiotic Use On Their Dams (Day before Calf Birth)
Once daily sampling (Experiment 1)	1	Jersey	–	–	–
	2	Holstein	–	–	–
	3	Holstein	–	–	–
	4	Holstein	–	–	–
	5	Holstein	–	–	–
	6	Holstein	–	–	–
	7	Holstein	–	–	–
	8	Holstein	–	–	–
	9	Holstein	–	–	–
	10	Holstein	–	–	–
Once weekly sampling (Experiment 2)	11	Holstein	–	–	–
	12	Holstein	–	–	–
	13	Holstein	–	–	–
	14	Holstein	–	–	–
	15	Holstein	–	–	–
	16	Jersey	–	–	–
	17	Holstein	–	–	–
	18	Holstein	–	–	–
	19	Holstein	–	–	–
	20	Holstein	–	–	–
	21	Holstein	–	–	–
	22	Holstein	–	–	–
	23	Holstein	Diarrhea (15, 19 d)	Ceftiofur (15 d)	–
	24	Holstein	Diarrhea (14–16 d) Diarrhea (21–25 d)	Ceftiofur (14–16 d) Ceftiofur (23–25 d)	–
	25	Jersy	Diarrhea (15–19 d)	Ceftiofur (15–18 d)	–
	26	Holstein	Diarrhea (16 d)	Ceftiofur (16 d)	–
	27	Holstein	Diarrhea (23 d)	Ceftiofur (23–24 d)	Ceftiofur (156 d)
	28	Holstein	–	–	Ceftiofur (329 d)
	29	Holstein	–	–	Ceftiofur (95 d)

Experiment 2 Ceftiofur was administered by intramuscular injection at a dose of 100 mg/kg body weight; Clinical signs of the corresponding dams were hoof disease (no. 27), foot rot (no. 28), and mastitis (no. 29).
